# Management of Oromandibular Dystonia: A Case Report and Literature Update

**DOI:** 10.1155/2017/3514393

**Published:** 2017-06-19

**Authors:** Suma Gn, Adrita Nag

**Affiliations:** ^1^Department of Oral Medicine & Radiology, SGT Dental College, Hospital and Research Institute, Gurgaon, Haryana 122505, India; ^2^Department of Oral Medicine and Radiology, Faculty of Dental Sciences, SGT Dental College, Hospital and Research Institute, Gurgaon, Haryana 122505, India

## Abstract

Oromandibular dystonia (OMD) is a movement disorder characterized by involuntary, paroxysmal, and patterned muscle contractions of varying severity resulting in sustained spasms of masticatory muscles, affecting the jaws, tongue, face, and pharynx. It is most commonly idiopathic or medication-induced, but peripheral trauma sometimes precedes the condition. We present a case report of a 26-year-old female patient who suffered repetitive bouts of hemifacial muscle contractions for 2 years on closing the mouth which interfered in patient's well-being and quality of life by hampering her ability to eat and talk and to the extent of inability to breath due to contractions of her neck muscles. Prompt diagnosis of a chronic oromandibular dystonia jaw closing type led to the control of the spasmodic muscle contractions within 24 hours and alleviation of patients fear of morbidity.

## 1. Introduction

OMD is considered as a focal dystonia involving mouth, jaw, and tongue, manifested by involuntary muscle contractions producing repetitive, patterned movements of the involved structures. Dystonia is either idiopathic (primary) or follows a peripheral injury. Head and neck dystonia manifest clinically by the presence of involuntary sustained, forceful muscle contractions, and characteristic rhythmic movements and abnormal posture. Craniocervical manifestations of dystonia affect the person's quality of life by interfering with the ability to speak and swallow and in social interaction. Dystonia is the most common prevalent movement disorder next only to Parkinson's disease (PD) and essential tremor (ET). Primary dystonia is common with a prevalence of 3.4 per 100,000 as generalized dystonia [1].

## 2. Case Report

A 26-year-old female patient visited the Department of Oral Medicine and Radiology with a chief complaint of spontaneous, painful constrictive movements on her right side of face with a feeling of constriction in her neck leading to difficulty in breathing. Patient was apparently normal 2 years back when she experienced spontaneous, intermittent, unilateral paroxysmal, severely painful involuntary spasmodic contractions on the right half of face which lasted for 3–5 minutes, repetitive throughout the day and which relieved on conscious opening of mouth to reappear again on next occlusal contact. The symptoms were distributed along the right half of forehead region, involving same side jaw up to right half of her neck. The neck constrictions were also associated with spasms internally in throat area causing difficulty in breathing. During these episodic contractions her jaws involuntarily closed and her tongue deviated towards the opposite side, with slurring of speech. The painful contractions were triggered while brushing, eating food, touching on right side of face, and excessive talking, breathing. During these episodic contractions her jaws involuntarily closed and tongue deviated to the opposite side with slurring of speech and episodes of accidental tongue bite on several occasions. Grimacing, reddening, and breathlessness were the associated findings. Her past dental and personal history was unremarkable except for a history of assault on the same side of face in the form of domestic violence. Her dental history reveals extraction of 48 six months back which was uneventful with normal postsurgical healing period. She had no history of consanguineous marriage and had no first-degree relative with neurological disorders. Patient reports multiple attempts towards treatment by various specialists including ENT and Psychiatrist, who prescribed her tricyclic antidepressants, gabapentin, with no cure; on the contrary she was branded as a psychiatric patient. Stressful events in her everyday life made the symptoms worse. The patient felt being rejected, sad, frustrated, depressed, and even anxious because the painful symptoms remained undiagnosed for a long time. The patient also experienced symptom-related depression, anxiety, and insomnia, which also created much anxiety among her family members.

Extraoral examination was marked with spontaneous fasciculations with respect to right side of face, with an appreciable bulge associated with diffuse swelling and redness. On intraoral examination the episodes began with fine involuntary fasciculations in the right masseter and temporalis which progressed to severe dystonic contractions of the face and neck within few seconds causing grimacing of the face with difficulty in breathing following which the patient assumed a body posture holding the right side of face and neck gasping for breath, trying to open her mouth in an attempt to breath. Once she forcefully opened her mouth the spasms would reduce within a span of 2-3 seconds and eventually stopped. Her TMJ examination revealed an anterior disc displacement without reduction; fine fasciculations were observed in her right side masseter and temporalis region. Marked reddening and an observable bulge were appreciated on the right half of forehead and jaw region. Brain MRI did not show any definite abnormal brain findings or brainstem lesion. Consultation with the neurologic department ruled out other neurologic disorders and this was confirmed by the absence of any other accompanying neurologic deficits. The cranial nerve examination was unremarkable. Hemifacial involuntary spasmodic contraction of masseter and temporalis was seen producing repetitive pattern of jaw closing pattern and tongue movements.

On palpation frank fasciculations were appreciated along the body of masseter and anterior and posterior bands of temporalis. Mild fasciculations were felt along the muscles of neck. These movements were more pronounced during clenching of posterior teeth and speech and chewing movements. The dystonic movements diminished with oral sensory feedback such as voluntary opening of mouth by the patient in an attempt to breathe through mouth.

**Figure 1:**
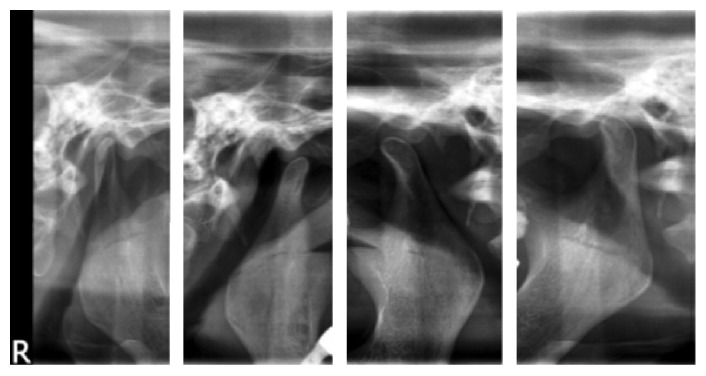
TMJ tomographic views which revealed an excessive anterior movement of the condyle on open mouth position.

**Figure 2:**
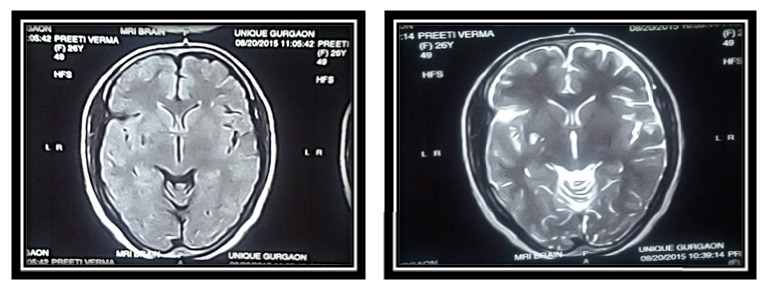
MRI brain scan reveals no focal pathology.

Investigations included assessment of temporomandibular function with TMJ tomographic views which revealed an excessive anterior movement of the condyle on open mouth position (Figure 1). MRI brain scan (Figure 2) revealed no focal pathology. Blood investigations were done for serum calcium levels to rule out hypocalcemic tetany which revealed parameters in normal range.

## 3. Differential Diagnosis

Based on her history and physical presentation, the differential diagnosis included psychogenic facial spasm, tardive dyskinesia, or oromandibular dystonia [2] with associated masticatory muscular pain, facial motor seizures, and hypocalcemic tetany: facial myokymia; Tourette syndrome; facial motor seizures.

(1) Myokymia is an involuntary, spontaneous, localized quivering of a few muscles or a bundle of muscles but which is insufficient to move a joint, example involuntary eyelid muscle contraction, typically involving the lower eyelid; complete and visible movement of the jaws was taking place in the presenting case.

(2) Facial myokymia is a fine rippling of muscles on one side of the face and may reflect an underlying tumor in the brain stem, for example, a brain stem glioma and loss of myelin in brain stem associated with multiple sclerosis. No focal pathology was detected in MRI of brain (Figure 2).

 (3) Tourette syndrome;* Tourette* or* TS* is an inherited neuropsychiatric disorder with onset in childhood, characterized by multiple physical motor tics and at least one vocal (phonic) Tic. These tics, characteristically wax and wane, can be suppressed temporarily and are preceded by a premonitory urge. Tourette is defined as part of spectrum of Tic disorder, which includes provisional, transient, and persistent (chronic) tics. Tic disorders in school-age children are higher, with the more common tics of eye blinking, coughing, throat clearing, sniffing, and facial movements. Extreme Tourette in adulthood is a rarity; (4) tardive dyskinesias (TDs) are involuntary movements of the tongue, lips, face, trunk, and extremities that occur in patients treated with long-term dopaminergic antagonist medications; our patient suffered from the muscular contractions for one and a half years with no previous drug history.

**Figure 3:**
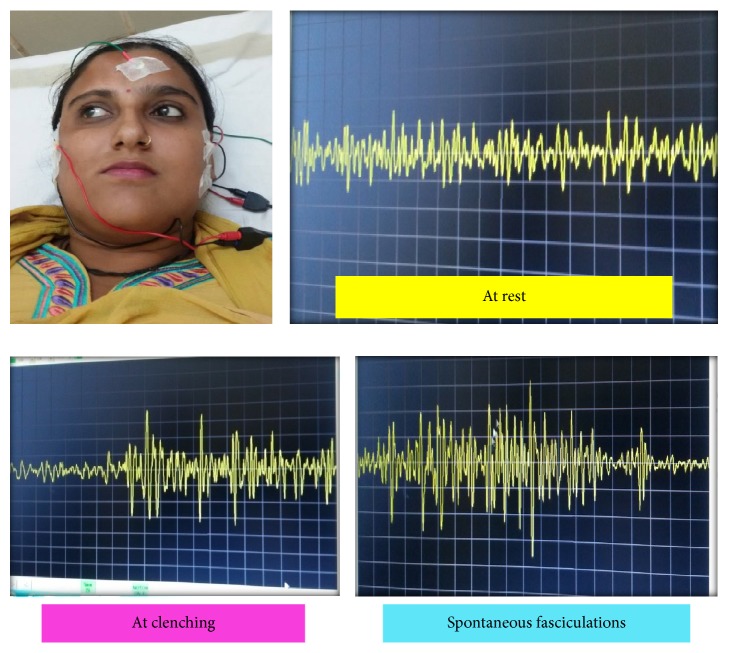
Electromyographic study of the bilateral temporalis and masseter reveals spontaneous fasciculations at rest.

To quantitatively assess the muscular contractions and to find the extent of muscle involvement electromyographic study of the bilateral temporalis and masseter was done. Electromyography activity was typically reflected as significant high frequency and high-voltage activity of motor unit potentials with either sustained or short-duration bursts of discharge patterns (fasciculations) at rest, which were normally electrically inactive [3] in the right side temporalis and masseter muscle (Figure 3).

**Figure 4:**
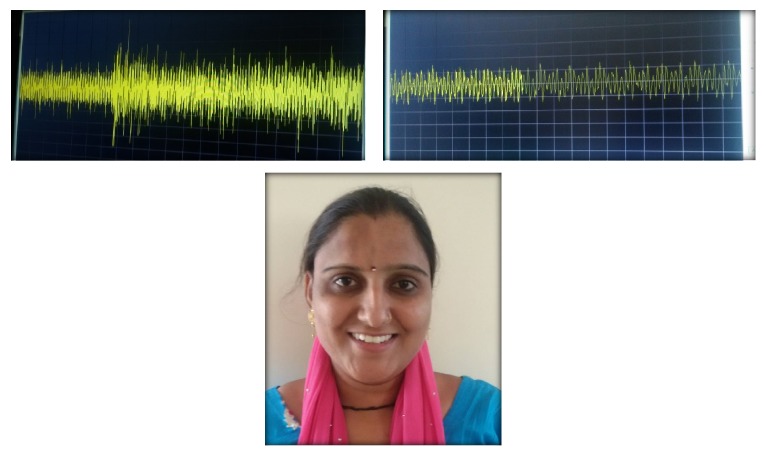
Follow-up visits at six-month period revealed complete absence of the dystonic contractions.

**Figure 5:**
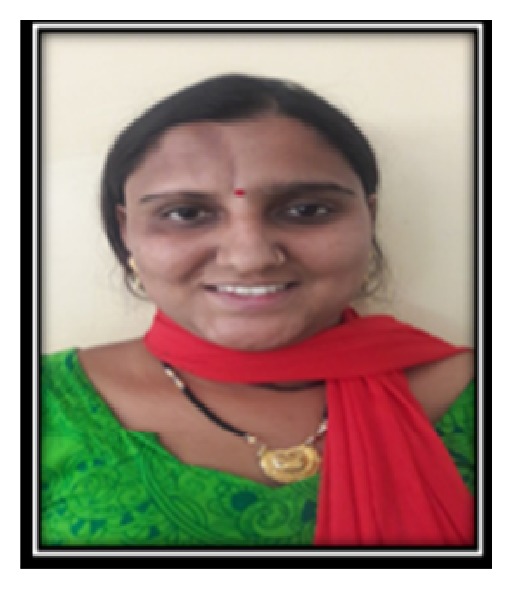
At 12-month recall, patient reports complete absence of dystonic movements with improved quality of life assessment with a maintenance dose of carbamazepine of half tablet at night time only. Patient reports to have been leading her normal life and was visibly happy during the checkup visit. However a marked bulge is still apparent on her right half of forehead region which could be due to the muscular hyperactivity with probable hypertrophy in the involved muscle, a feature which has not been reported in the previous reported literature.

Based upon the presenting clinical features, examinations and investigations for a working diagnosis of oromandibular dystonia (jaw closing) type were made. A differential diagnosis of facial myokymia, facial motor seizures, myoclonus, muscular spasms, and tardive dystonia was considered. Reassurance was the primary approach towards the treatment goal with a positive reinforcement of the curability of the disease. Patient was prescribed tablet Tegretol (carbamazepine) 200 mg BD dose and recalled after 3 days. She showed a definite reduction of the dystonic movement, becoming symptom free. No side effect was observed and patient was visibly happy and reported to have eaten a complete meal without any discomfort after almost a year. Patient was revaluated for the muscular functions and advised to continue with the medications. Follow-up after three months was done and revaluation of the muscular functions was done with electromyography study, which revealed absence of fasciculations, contractions and quality of life assessments were done, and a significant improvement in the assessment score was calculated (Figure 4). The dosage of carbamazepine was titrated to 200 mg OD once a day (Figure 5).

Tabular presentation of the features of the present case is enlisted in Table 1.

## 4. Discussions

**Figure 6:**
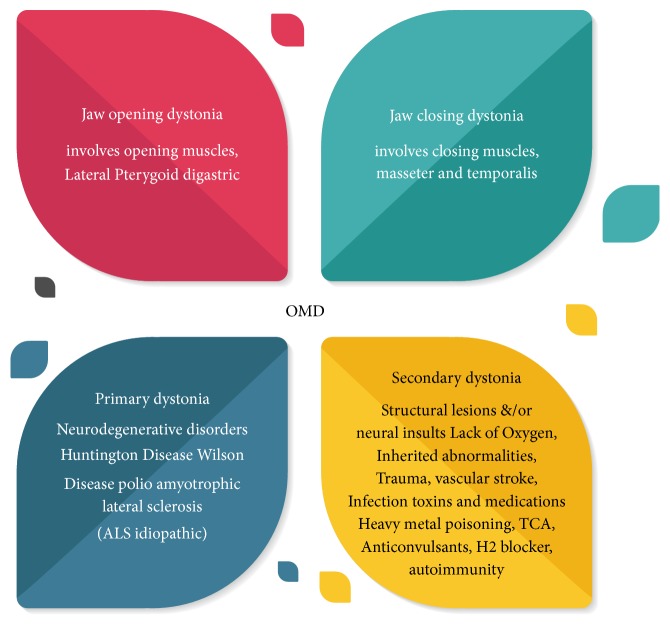
Classification of oromandibular dystonia based on etiology.

The terms oromandibular dystonia, craniocervical dystonia, or Meige syndrome describe a focal or segmental dystonia whereby repetitive sustained spasms of the masticatory, facial, or lingual muscles result in painful, involuntary, movement of the jaws. Oromandibular dystonia is a rare condition; misdiagnosis is common as it may mimic signs and symptoms of temporomandibular joint disorders or other movement disorders. The diagnosis of dystonia is challenging, as recognition of clinical findings at the time of presentation is affected by several factors such as the psychological status of the patient and the training of the clinician. Dystonia is classified as focal, segmental, multifocal, and generalized (Figure 6). Furthermore, it can also be classified into the affected body parts depending on anatomical regions of distribution [1, 4–6].

Another method for classifying dystonia is by etiology, into primary and secondary. Primary forms are also referred to as idiopathic, inherited, or familial.

**Figure 7:**
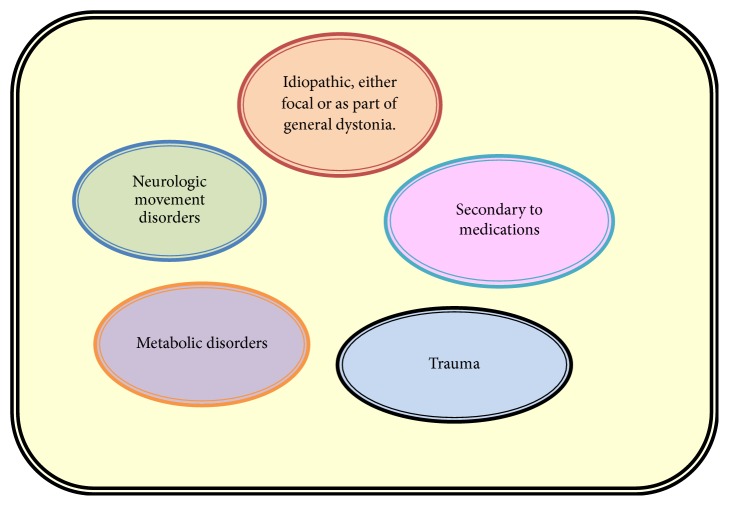
Etiology of Oromandibular Dystonia.

The electrophysiological data of these patients suggests that dystonia is associated with several changes in neuronal activity in striatal circuits such as an alteration in the rate, pattern, somatosensory responsiveness, and synchronization of neural activity in palladium thalamocortical circuits (Figure 7) [7]. However the relationship between changes in neural activity in these regions and the development of dystonia is still not clear [8].

Early detection of the patients complaint and having an understanding of the anatomy which is responsible for the characteristic clinical signs and symptoms play a significant role in successful management of the case [9, 10]. In our case the classic presentation of the patient in the form of spasmodic contractions with repetitive pattern triggered by occlusion of tooth indicated towards the jaw closing type of OMD. The exclusive involvement of the right side masseter and temporalis indicated a focal type of presentation. The involvement of the neck muscles on the right side resulting in the feeling of constriction suggests cervical component of dystonia, since there is no gold standard diagnostic test or biomarker for testing the validity of the diagnosis. The various therapeutic modalities which are promising in successfully controlling the symptoms are the therapeutic medications in the form of Botox injections, oral antidystonic therapies. Physical therapy modality including speech therapy, oral sensory devices and biofeedback, and so forth also have a positive role. Both jaw opening and jaw closing OMD can be treated with oral antidystonic therapies such as tetrabenazine, diazepam, and carbamazepine. Anticholinergic drugs reduce muscle spasm by centrally inhibiting the parasympathetic system. Benzodiazepine decreases monosynaptic and polysynaptic reflexes by increasing presynaptic GABA inhibition a similar action to Baclofen. Anticonvulsants such as carbamazepine reduce severe muscle spasm by decreasing polysynaptic response [11].

**Figure 8:**
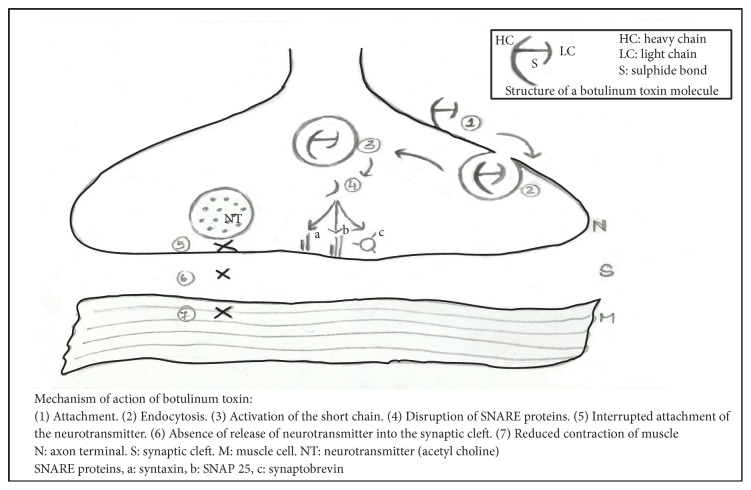
Mechanism of action of botulinum toxin.

*Role of Botulinum Toxin in Oromandibular Dystonia.* The botulinum toxin (BTX) is a naturally occurring neurotoxin that is produced by gram-positive anaerobic bacteria* Clostridium botulinum. *Botulinum toxin type A (BTX-A) is the most commonly used form which is prepared by Hall strain* Clostridium botulinum fermentation*. A standard vial of BTX-A contains 100 units of toxin, 0.5 mg of human albumin, and 0.9 mg of sodium chloride. For jaw opening dystonia the lateral pterygoid muscle is injected with 45 units of BTX-A, by an intraoral injection approach following the ramus of the mandible to locate the lateral pterygoid and injecting approximately 45 units on each side. For jaw closing dystonia, BTX-A is injected into the masseter muscle at the angle of the mandible and 20 units of BoNT are injected into each site. Botulinum toxin acts on neuromuscular junction through the steps of (1) attachment, (2) endocytosis, (3) activation of short chain, and (4) disruption of SNARE proteins as depicted in Figure 8.

Adverse effects reported are dry mouth, dysphagia, lethargy, generalized weakness, and dysphonia. Relative contraindications include pregnancy, lactation, neuromuscular diseases, motor neuron disease, and concurrent use of aminoglycosides.

## 5. Mechanism of Action of Botulinum Toxin

Many cases of orofacial dystonia's after dental procedures have been reported; Sankhla et al. [12] reported 27 peripherally induced OMD, four of which were wearing new sets of dentures, including one patient with an ill-fitting dental bridge. Among the patients with ill-fitting dentures a habit of manipulation of the jaw muscles to stabilize the new dentures was observed. A possibility of proprioception impairment of the oral cavity leading to subsequent development of dystonia or so-called “edentulous dyskinesia” was proposed [13]. Hamzei et al. [14] reported the case of a woman who developed facial dystonia within a few hours and severe life-threatening laryngeal dystonia with respiratory failure within 3 days after insertion of ill-fitting dentures [13].

## 6. Conclusion

The Ultimate burden on oral health is of significant interest to the dentist as a vast range of dental implications are reported in the past literature in the form of Attritions, TMJ dysfunctions, increased cares risk, denture instability, loss of multiple teeth, alveolar atrophy, damage to restorations, and marginal to advanced periodontitis. It is important for the dentist to be familiar with oromandibular dystonia, as it can develop after dental treatment. Very few reported cases in Indian population exist as often these disorders are labeled psychogenic or characterized as temporomandibular disorders. As dentists, our main aim and goal would be to identify such often misdiagnosed cases of suffering patients often pushed to the realm of mental illness as many a time we might be their only hope. Prompt diagnosis in the presented case formed the key to a successful management and improved the quality of life of a disheartened patient.

## Figures and Tables

**Table 1:** Clinical features and investigations in the diagnostic work-up of the case.

Sl. number	Age	Clinical features	History	Investigations	Diagnosis	Treatment and follow-up
1	27/F	Spontaneous, intermittent, unilateral paroxysmal, severely painful involuntary spasmodic contractions; spasms internally leading to difficulty in breathing	No relevant family history	TMJ tomographic projections, electromyography (pre- and posttreatment evaluation), CT brain, Blood investigations	OMD (jaw closing type, primary dystonia)	Carbamazepine BD dose with 1, 3, 6, 9 months' follow-up with patient testimony
